# The origin of the recent (2012–2016) seismic activity in the Guadalajara, Jalisco, Mexico, area: A block boundary interaction?

**DOI:** 10.1371/journal.pone.0200991

**Published:** 2018-08-30

**Authors:** Jaime Yamamoto, Juan M. Espíndola, A. Zamora-Camacho, Guillermo Castellanos

**Affiliations:** 1 Department of Seismology, Instituto de Geofísica, Universidad Nacional Autonoma de Mexico, Mexico City, Mexico; 2 Department of Vulcanology, Instituto de Geofísica, Universidad Nacional Autonoma de Mexico, Mexico City, Mexico; 3 Department of Exact Sciences, Centro Universitario de la Costa, Universidad de Guadalajara, Guadalajara, Jalisco, Mexico; 4 Department of Project Engineering, Centro Universitario de Ciencias Exactas e Ingenierias, Universidad de Guadalajara, Guadalajara, Jalisco, Mexico; Royal Holloway University of London, UNITED KINGDOM

## Abstract

The central part of Jalisco, Mexico, has experienced low-magnitude earthquake sequences and swarms. Although the effects of these earthquakes have been limited to relatively small areas, the earthquakes have caused general alarm among the population and, in some cases, have been catastrophic. These earthquake swarms are significant because they affect the most populous area of the state, including the capital city of Guadalajara. An extraordinary example is an earthquake swarm that started on 8 May 1912 and lasted until September of that year. The region remained seismically quiescent until May 2012, when seismic activity resumed, lasting to the present. We analyze the recent seismic activity, starting with the earthquake of 18 May 2012 (03:07 UT) at the western edge of Lake Chapala and ending with the magnitude 4.2 earthquake on 3 November 2016. Our analysis includes eight earthquakes with magnitudes between 3.5 and 4.8, the revision of hypocenter locations, and the determination of focal mechanism solutions using the inversion of the moment tensor method. When possible, inversion solutions are compared with solutions obtained with the first arrival polarity method. We compare our results for the recent seismicity with the distribution of reported damage associated with historical earthquakes. Our work indicates a N-S trending seismic source zone and an orientation of nodal planes that suggests reactivation of preexisting local faults induced by the interaction of the western border of the Trans-Mexican Volcanic Belt with the eastern border of the Jalisco Block.

## Introduction

**Fig 1:**
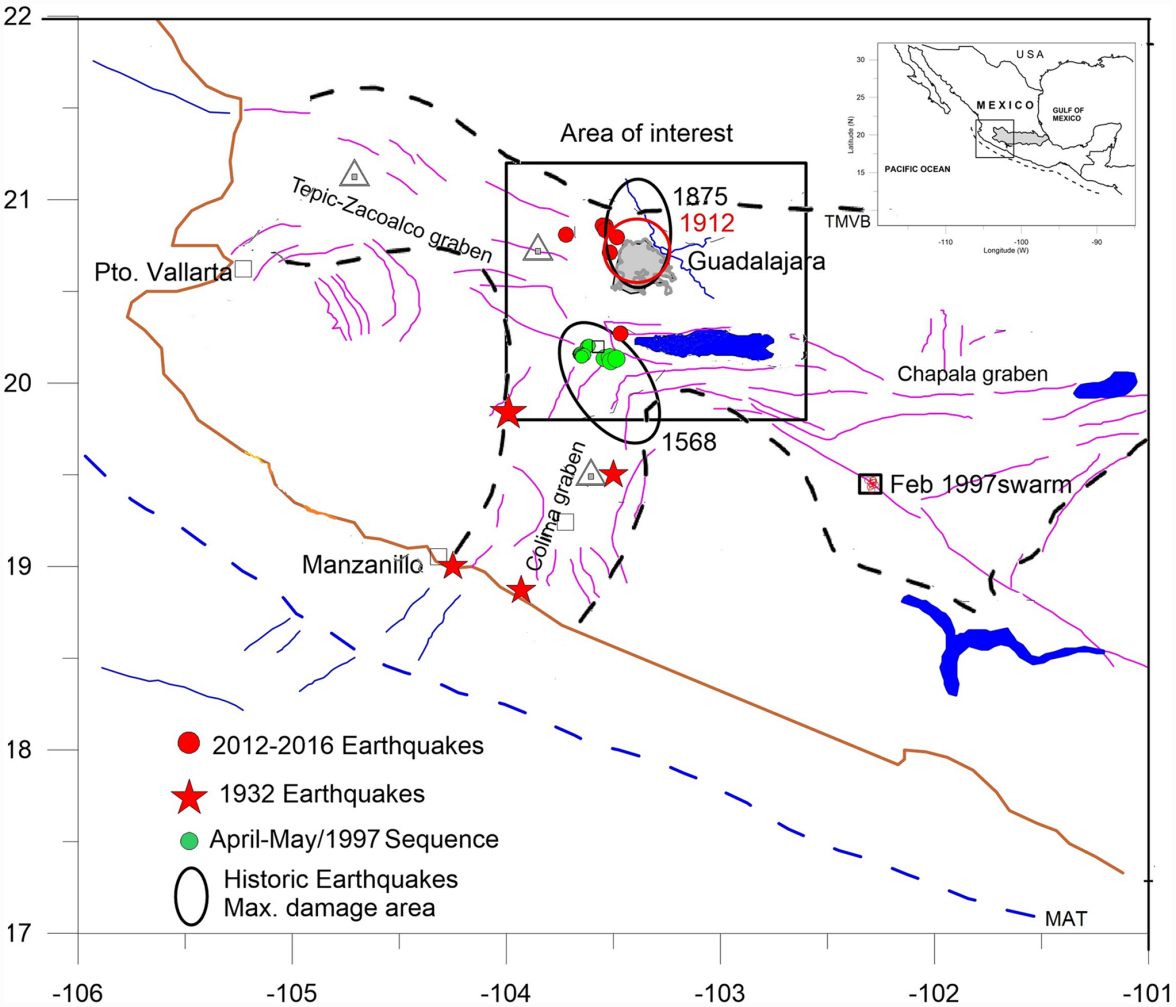
Map of the region under study. JB indicates de Jalisco Block. Ellipses mark the areas of maximum damage observed during the 1568 and 1875 earthquakes and the 1912 earthquake sequence. Red stars are major earthquakes in 1932. Red circles are the earthquakes analyzed in the present study. Green circles are epicenters of the 1997 earthquake sequence. Triangles are active volcanoes. Line segments are faults and fractures. Blue and bold dashed lines are the Middle America Trench and the Trans-Mexican Volcanic Belt limit, respectively.

Central western Mexico is one of the most complex geotectonic regions of the country owing to the convergence of several geological structures whose interactions give rise to a complex seismic pattern [1]. Seismicity consists mostly of shallow to intermediate-depth earthquakes. The focal mechanism solutions are predominantly of reverse type at the coast and normal type inland and are attributed to the subduction of the Rivera and Cocos plates under the North America Plate [2]. In the region, low-magnitude earthquakes occur frequently in the form of upper-crust shallow-depth sequences and swarms, which have been associated with the fractured nature of the Trans-Mexican Volcanic Belt (TMVB) [3, 4] (Fig 1). Even though these low-magnitude earthquakes should not represent a high danger to present-day construction in the region, their proximity to population centers could cause considerable local damage, and psychological effects on the population cannot be understated. Besides, historical evidence indicates that major inland earthquakes have occurred sporadically in the past.

The population of the state of Jalisco (7.4 million) ranks fourth in Mexico, and the state capital of Guadalajara, home to approximately 1.5 million inhabitants, is showing rapid economic growth. Historical records reveal that a major (*M*_*w*_ >7) earthquake occurred on 27 December 1568, southwest of Guadalajara near the northeastern corner of the Jalisco Block (JB) [5]. In 1875, a catastrophic earthquake struck the town of San Cristobal, located 40 km north of Guadalajara [6]. A better understanding of crustal seismicity in this populated region is essential.

## Geotectonics of western Mexico: Overview

Western Mexico is tectonically dominated from west to east by the subduction along the Middle America Trench (MAT), a region of extension marked by the rift system bounding the JB and the TMVB [7]. The western extreme of the TMVB presents neotectonic features that define the JB [7, 8]. The JB basement consists of two geological provinces, both partially covered by rocks belonging to the TMVB. The Tepic-Zacoalco graben, Colima graben, and Chapala graben located to the north, south, and east, respectively, define the limits of the JB structure (Fig 1). The TMVB origin has been related to northeastward subduction of the Cocos and Rivera plates beneath the North America Plate along the MAT. The general E-W orientation of the volcanic belt as defined by the Colima-Popocatepetl-Citlatepetl volcano chain diverges from the trend of the MAT at the Pacific Coast. This divergence has been explained by an eastward increase in convergence rate and an eastward shallowing of the Benioff zone [9].

The TMVB is a complex continental arc 1000 km long extending from the western volcano of San Juan to the eastern Gulf of Mexico coast. Its crust is approximately 35–40 km thick in the west and becomes thicker to the east. Tectonic and volcanic activity in central Mexico has produced numerous volcanic centers, grabens, and crater lakes. Many of these tectonic and volcanic depressions are occupied by both graben and volcanic lakes. The Lake Chapala basin is located in the E-W-trending Citala rift, which forms the so-called Jalisco continental triple junction together with the Tepic-Zacoalco and Colima rift. Volcanic sedimentary deposits at the Lake Chapala basin and at the Zacoalco and Colima tectonic depressions are approximately 900–1000 m thick and comprise a sequence of lacustrine sediments and pyroclastic units of ash and pumice [10].

## Historical seismicity of the region

Recorded evidence indicates the occurrence of several major earthquakes near the coast along the segment Jalisco-Northern Guerrero [11]. Of particular importance is the series of major earthquakes that occurred in the region in June 1932 (7 ≤ *M* ≤ 8.2). Fig 1 shows the region’s earthquakes that are most relevant to our study.

Most of these earthquakes are of shallow-depth focus located between the coast and the MAT. Focal mechanism solutions for these earthquakes are of reverse faulting type on a low-angle (~17°) plane resulting from subduction of either the Rivera Plate or the Cocos Plate under the North America Plate [12]. In most cases, the association of earthquakes to a particular regional geological unit in this area is difficult to establish because of the lack of well-defined separation boundaries. It has been accepted, however, that the series of major earthquakes of Jalisco-Colima in 1932 occurred along the interplate of the Rivera and North America plates [13, 14].

According to Suarez et al. [5], four large earthquakes occurred along the TMVB in 1568, 1875, 1912, and 1920, respectively. The first two earthquakes occurred near the city of Guadalajara, Jalisco, and are relevant to our study.

### Earthquake of 27 December 1568

Historical documents indicate that on 27 December 1568, a strong earthquake (*M*_*w*_ **>** 7) occurred at the southeastern border of the JB, probably at the junction of the Tepic-Zacoalco and Colima rifts near the western border of Lake Chapala. The position of the epicenter was estimated [5] using well-documented accounts of the earthquake´s damage to houses, churches, and Franciscan missions of the region and of the observed ground deformations, cracks, and landslides. Many churches located in villages along the Tepic-Zacoalco and Colima rifts suffered damage, and a total of 62 persons were reported dead. In Guadalajara, the earthquake was described as producing very strong ground shaking and damage to some buildings. Most of the damage from this event took place along the JB’s northern and eastern boundaries.

Fig 1 shows the areas in which damage was observed during the 1568 earthquake. Most areas of damage are located along the Tepic-Zacoalco rift and north of the Colima rift, with the largest intensities observed at the junction of these two geological provinces near the town of Zacoalco. Because the area is strewn with faults [15], mostly of normal type, with the available information, it was not possible to associate the 1568 earthquake to a specific fault.

### Earthquake of 11 February 1875

On 11 February 1875 occurred a strong earthquake, probably *M* > 7, at 20:23 LT that produced heavy damage to the towns of San Cristobal and Guadalajara [16]. The occurrence of this event initiated a sequence of earthquakes that kept the populations of Guadalajara and nearby towns in suspense for several months. According to inhabitants’ descriptions, the main shock was felt in Guadalajara as a “furious shake accompanied by underground sounds” and mostly up-and-down motion. In San Cristobal, a “noise like thunder” was also heard by the frightened population. The entire village of San Cristobal came down in one single onslaught; 26 persons lost their lives there. Fig 1 shows the area where damage was reported.

### Earthquake sequence of 8 May 1912

On 8 May 1912, at 06:36 LT, an earthquake sequence was initiated near the area of a previous earthquake sequence in 1875 [17]. The next day, on 9 May, a similar earthquake was felt. There was general alarm in the capital city of Guadalajara (which had approximately 100,000 inhabitants in 1912) and nearby towns, as the earthquake opened up cracks, threw down effigies of saints in the churches, rang bells in church towers, and detached plaster from walls. The ground motion was intense, with marked up-and-down motion of short duration. Over the next 24 hours, three more earthquakes were felt, and the activity lasted until the end of September of the same year. During the first 18 days, 64 tremors were felt. Ordoñez [17] mentions that the affected area was very small and can be framed in an 18 × 18 km square, which would include the towns of San Pedro Tlaquepaque and Zapopan.

### Earthquake swarm and sequence of 1997

More recently, Pacheco et al. [3] reported an earthquake swarm during February and March 1997 along the Chapala-Oaxaca fault zone; 230 earthquakes in the magnitude range of 1.5–3.5 and with focal depths from 10 to 18 km were located. Their epicenter distribution shows a N-E alignment. The fault plane solution of the best recorded earthquakes shows a left lateral strike-slip motion with a normal component. According to the authors, the preferred fault plane strikes N-E and coincides with the alignment of events, indicating the preexistence of local faults.

Moreover, Pacheco et al. [4] also analyzed an earthquake sequence in the southeastern tip of the Tepic-Zacoalco graben during the months of April and May 1997. A total of 33 events in the magnitude range of 1.5–3.5 were recorded and located using a portable array of seismographs. According to the authors, two groups were undoubtedly identified: one shows shallow normal fault solutions with one nodal plane striking NW-SE and dipping steeply toward the NE, and another shallow plane dips to the SW. A third group of observed events can likely be associated with a deeper fault system.

## Recent seismic activity in the region

**Fig 2:**
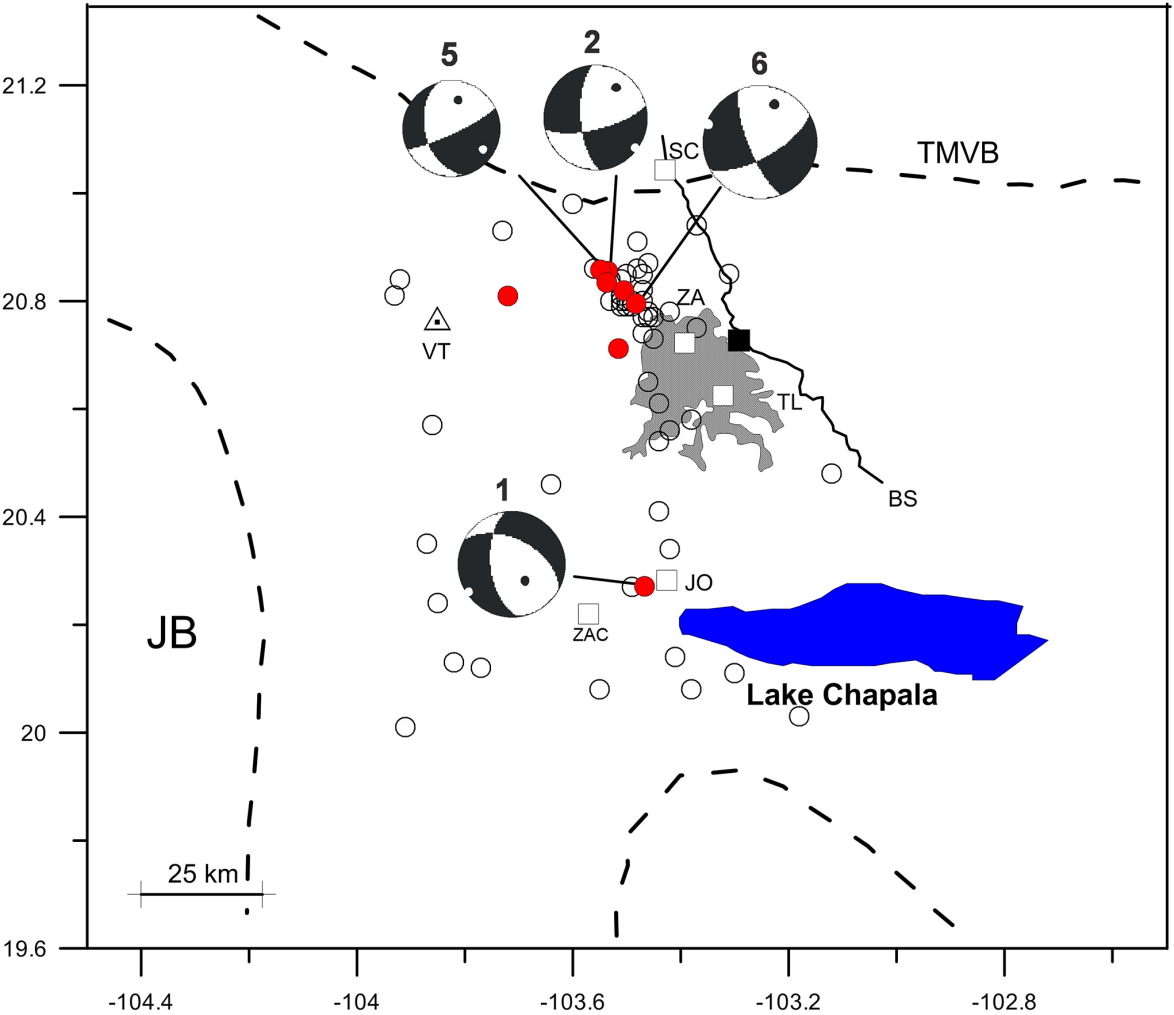
Relocation of the studied earthquakes. The map shows with shading the Guadalajara metropolitan area. Open red circles are the epicenters and the fault plane solutions of the earthquakes analyzed. Focal spheres contain the nodal planes of the preferred solutions with the minimum RMS errors. The numbers correspond to the events listed in Table 1. The overall seismic activity (4.0 ≤ *M* ≤ 4.5) for the 2006–2016 period as reported by the Servicio Sismologico Nacional of Mexico (SSN) is shown with black open circles. VT is the Tequila volcano. JO, TL, ZA, SC and ZAC are the Jocotepec, Tlaquepaque, Zapopan, San Cristobal and Zacoalco towns respectively. BS is the Barranca de Santiago. TMVB marks the limit of the Trans-Mexican Volcanic Belt. The black square shows the position of the temporary seismic station in operation from 2015 to 2016. Events 1 and 6 are the larger-magnitude earthquakes of the analyzed sequence.

Fig 2 displays the overall local seismic activity (4.0 ≤ *M* ≤ 4.8) in the zone of interest for the period 2006–2016. The figure shows a seismic activity pattern with a nearly N-S trend that clusters near the northwest corner of the Guadalajara metropolitan area.

**Table 1:** List of analyzed earthquakes.

No.	Date	Time	Lat. (°N)	Long. (°W)	Depth (km)	*M*	Stat.	Dis. range (km)	Δlat (km)	Δlong (km)	Δdepth (km)	RMS (s)
1	18 May 2012	3:07:55	20.271	103.467	15.20	4.5	18	11–531	4.8	4.2	5.3	1.22
2	15 Dec 2015	16:09:21	20.855	103.535	12.70	4.4	23	29–542	6.1	5.6	11.4	1.84
3	15 Dec 2015	16:32:33	20.809	103.720	18.20	3.6	9	21–236	5.0	8.8	8.0	0.98
4	15 Dec 2015	17:49:44	20.858	103.549	15.00	3.9	9	31–422	9.6	17.8	16.8	1.93
5	17 Dec 2015	7:59:09	20.834	103.537	11.90	4.1	23	28–576	6.7	5.6	11.7	1.97
6	11 May 2016	22:35:16	20.795	103.483	10.60	4.8	65	13–1182	3.4	3.2	5.5	1.70
7	28 Jul 2016	18:21:02	20.712	103.515	8.00	3.9	16	23–512	6.0	5.2	9.0	1.48
8	3 Nov 2016	06:15:14	20.810	103.492	10.7	4.2	23	23–630	4.7	4.3	8.2	1.50

*Note*. Times (H:M:S) are in UT. Stat. and dis. range indicate the number of the seismic stations utilized and the range of distances, respectively; Δlat, Δlong, and Δdepth are the latitude, longitude, and focal depth errors, respectively.

From the earthquakes plotted, two events (Event 1 and 6 in Table 1) stand out: the 18 May 2012 (*M* 4.5) Lake Chapala earthquake and the 11 May 2016 (*M* 4.8) Guadalajara earthquake, which have larger magnitudes than the other earthquakes analyzed. Both were widely felt in the region, producing slight damage in Guadalajara and other nearby towns.

## The 18 May 2012 Lake Chapala earthquake

On 18 May 2012 at 03:07 UT (22:07 LT of 17 May), a 4.5 magnitude earthquake at 20.27°N, 103.467°W occurred at 15 km depth. The epicenter was located near the western margin of Lake Chapala and was widely felt throughout the state of Jalisco, including in the Guadalajara metropolitan area. According to the media, approximately 44,000 persons from 163 buildings were evacuated in the downtown area and other zones of the city as a safety measure. Minor damage to construction, such as broken glass windows and cracks and fissures to walls, was reported. Inhabitants of the city described the initial earth motion as a short, strong “push,” and then the amplitude of the motion increased with an up-and-down motion. In Zapopan, underground sounds were heard, and in the area of Tlaquepaque, the earthquake was felt strongly, although no damage was reported. Jocotepec, a town close to the epicenter, reported fissures in the ground and minor damage. The earthquake was widely felt north and south of Guadalajara and the northern margin of Lake Chapala.

## The 11 May 2016 Guadalajara earthquake

An earthquake occurred at 22:35 UT (17:35 LT) on 11 May 2016 near the northwestern limit of the Guadalajara metropolitan area. In Guadalajara, the earthquake caused a disruption in the electrical supply, generating a large vehicle traffic jam and prompting the evacuation of many buildings, although the damage reported was minor [18].

In several buildings in Guadalajara and Zapopan, cracks and fissures in wall plaster were reported. Some brick fences collapsed during the earthquakes. Telephone and electrical services were temporarily suspended. As a measure of precaution, the urban train service was also suspended. A person was injured by a falling rock, and many others with nervous crises were transferred to hospitals for medical attention.

## Seismic data analysis

The importance of studying the seismic characteristics of the Guadalajara area become apparent from the above mentioned. In the following subsections we analyze broadband seismic data available and present some useful results for the proper seismic risk assessment of the area.

From the data available, eight earthquakes were selected for analysis, because these earthquakes were recorded by the permanent stations of the SSN and Jalisco seismological networks and a portable station installed in the outskirts of Guadalajara near the Barranca de Santiago.

In Table 1 and Fig 2, we list the focal parameters of the earthquakes analyzed in this article and plot their positions. The locations were computed using arrival times of *P* and *S* waves recorded by the permanent and portable seismic stations (short-period and broadband). After testing several velocity models, we decided to use the one proposed by Pacheco et al. [4], because it produced lower residuals and RMS values. The number of stations used in the location procedure varies from event to event, and the resultant values of the location errors and RMS can be seen in Table 1. All the computations were carried out with the SEISAN seismic analysis package [19]. The quality of the arrivals indicates that the locations of the earthquakes are reliable. The calculated hypocenter depth is less precise but reliable, because according to our results, the errors are on average ±9.7 km owing to the fact that we used data from a seismic station close to the epicenters. Moreover, to ensure a correct depth determination, graphs of RMS versus depth for each event were drawn, and the focal depth calculated by the SEISAN program was cross-checked with the minimum of the graph. The final results are listed in Table 1. Magnitude M values in table are taken from SSN Internal Reports [20].

## Fault plane solutions and their interpretation

The fault plane solutions were obtained through the inversion of the moment tensor (CMT) with the procedure proposed by Dreger and Helmberger [21] and implemented in the SEISAN package. The method was tested thoroughly in Dreger and Helmberger [22]. The method assumes that we know the velocity model; using an inappropriate model will have significant effects on the final result. Also, Dreger and Helmberger recommend using at least four three-component stations to ensure better results. The goodness of the adjustment between the synthetic and the observed seismograms is calculated through a quality factor (*Q*) that goes from 0 to 4, with 4 corresponding to an optimal adjustment.

In our analysis, the quality of the inversion varies from event to event. The final results are shown in Table 2. General comments for each solution follow.

**Table 2:** Fault plane solution parameters as obtained from the inversion.

No.	Az 1	Dip 1	Rake 1	Az 2	Dip 2	Rake 2	P az	P dip	T az	T dip	*M*_*o*_ (N-m)	*M*_*w*_
1	177	54	−59	312	46	−125	146	65	246	4	2.9 × 10^14^	3.6
2	176	66	−159	77	71	−26	35	31	127	3	5.6 × 10^14^	3.8
5	165	44	−166	65	80	−47	13	39	123	23	2.4 × 10^14^	3.5
6	158	73	−156	61	67	−18	21	29	289	4	5.5 × 10^15^	4.5

*Note*. Az, dip, and rake are the parameters of the nodal planes. Numbers 1 and 2 correspond to the two nodal planes. P dip and P az are the dip and azimuth of the pressure axis, and similarly for the tension axis. Parameters are expressed in degrees. *M*_*o*_ is seismic moment of the earthquake; *M*_*w*_ is moment magnitude.

For Event 1, the accepted inversion was obtained using records from six broadband stations. Assuming that Plane 1 in Table 2 corresponds to the rupture plane, the solution indicates a normal mechanism with a left lateral component along a nearly N-S rupture plane dipping 54° to the west.

**Fig 3:**
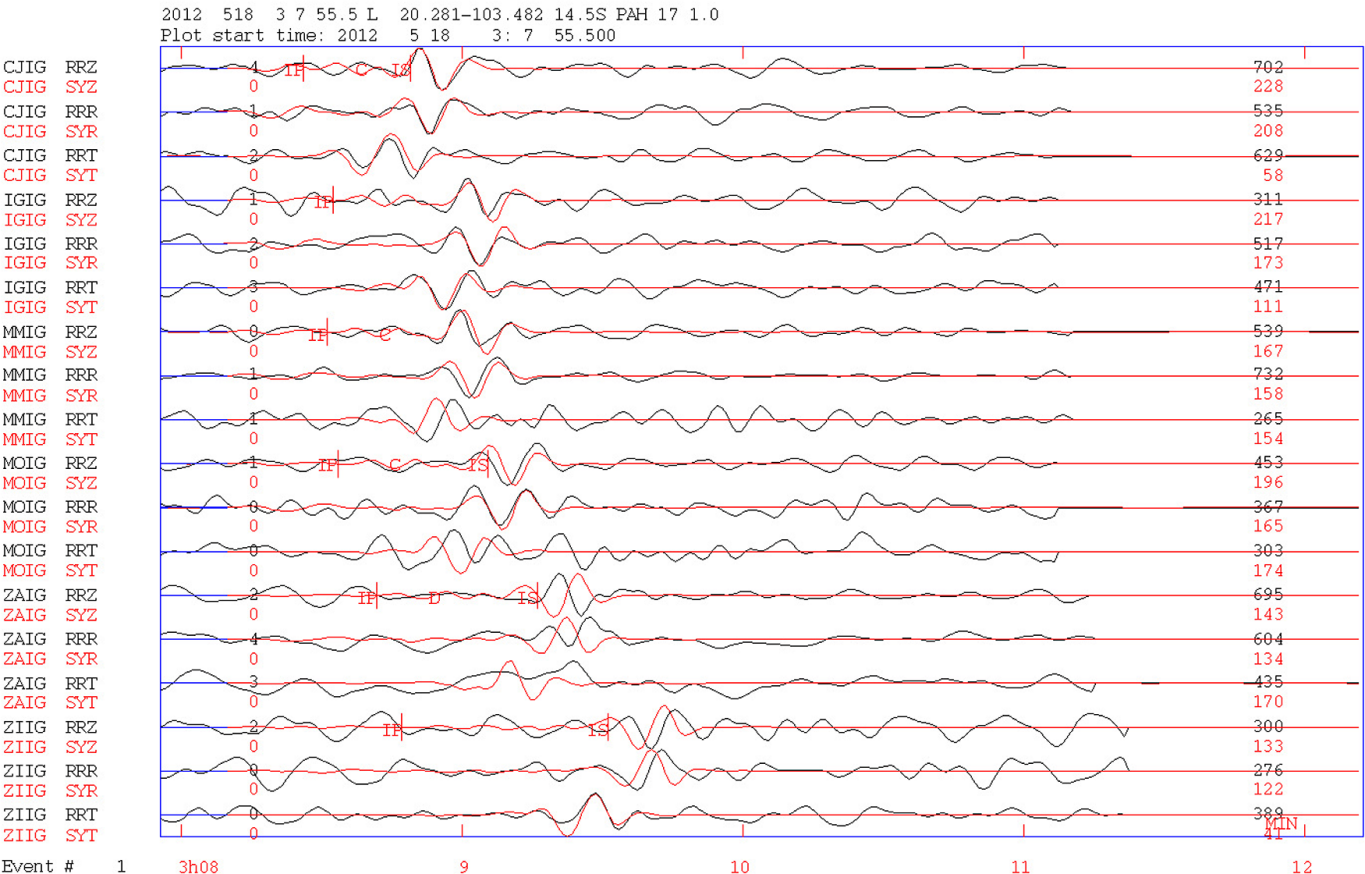
Seismic tensor inversion for Event 1. Plot of original filtered (0.01–0.1 Hz) and instrument-corrected data compared to the synthetic seismograms. Model and observed signals are shown with red and black lines, respectively. Station and component codes are shown in the first and second columns, respectively. RRZ is the vertical component. The original horizontal component seismograms (N-S and E-W) are rotated to obtain the radial (RRR) and transversal (RRT) components shown in the figure. For display reasons, the data are autoscaled, and in the right column, the amplitudes of the observed and synthetic signals are shown. The nearest station, CJIG, is 185 km from the epicenter, and ZIIG, the farthest, is 364 km from the epicenter.

In Fig 3, we show the waveform inversion result using data from six broadband stations for Event 1. Even though the quality of the theoretic and observed signal adjustment as reported by the program is poor (*Q* = 0), the fitting appears visually acceptable. Additionally, the resultant fault plane chosen as the rupture plane direction (N-S) is consistent with the one obtained by the polarity of the first arrivals (see S1 Fig).

The Event 1 of 18 May 2012 was a shallow-depth earthquake (15 km) that occurred in the western flank of Lake Chapala at the junction of Tepic-Zacoalco, Colima, and Chapala grabens. It should be mentioned that the epicenter location falls near the site at which Pacheco et al. [4] recorded and located the 1997 earthquake sequence. Additionally, these authors reported a similar focal mechanism solution for the local earthquakes studied in their article.

**Fig 4:**
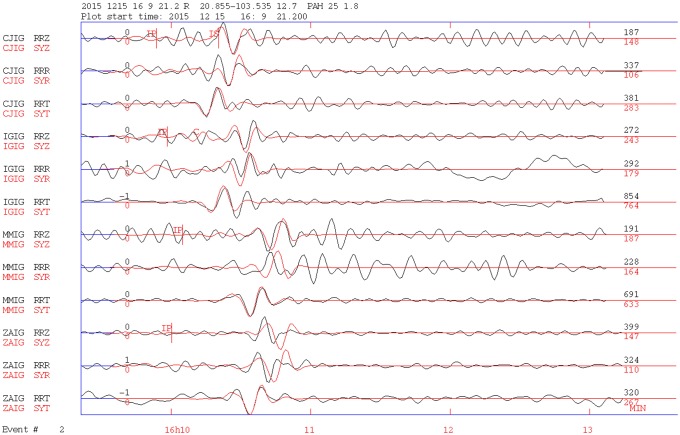
Seismic tensor inversion for Event 2. Theoretical and observed signals are shown with red and black lines, respectively. The inversion was carried out with four broadband stations. The nearest station, CJIG, is 218 km from the epicenter, and MMIG, the farthest, is 285 km from the epicenter. Other labels are as in Fig 3.

The Event 2 earthquake occurred near the northwestern tip of the Guadalajara metropolitan area about 60 km north of Event 1. The focal mechanism was obtained by inverting records from four broadband stations, and the result is shown in Fig 4. The visual adjustment of the observed and calculated signals looks quite well, even though the program reports *Q* = 0. The result indicates a normal faulting mechanism, with the rupture occurring along a plane striking nearly N-S. Its solution is similar to the solution of Event 1. The focal depths of both earthquakes are quite similar, at 13 km and 15 km, respectively.

**Fig 5:**
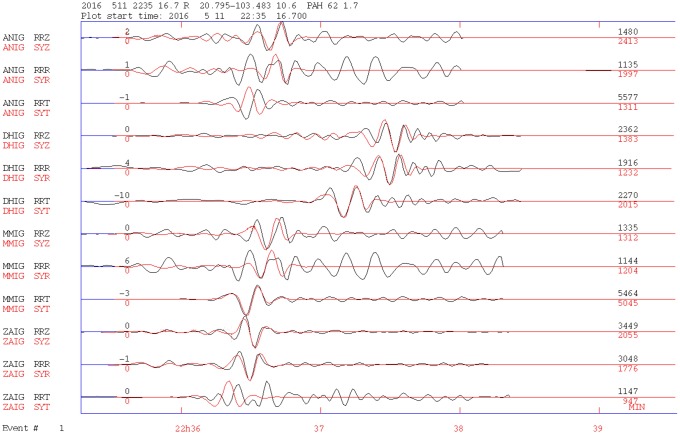
Seismic tensor inversion for Event 6. Theoretical and observed signals are shown with red and black lines, respectively. The inversion was carried out with four broadband stations. The nearest station, ANIG, is 108 km from the epicenter, and DMIG is the farthest at 467 km from the epicenter. Other labels are as in Fig 3.

Event 6 is also located to the northwest of Guadalajara at 11 km depth. This is the larger-magnitude (*M* 4.8) event analyzed in this article. Its fault mechanism was obtained by inverting four broadband stations, as shown in Fig 5. According to Dreger’s program fitting indicators, this earthquake is the best adjusted, with *Q* = 3. The solution indicates a faulting with a rupture plane striking in a nearly N-S direction. This result agrees with the solution obtained with polarities of the firsts’ arrivals (see S2 Fig).

Singh et al. [23] studied this earthquake using data from two accelerographs installed in the Guadalajara area. They obtained a rupture plane oriented N-NE and dipping 49° to the east. Singh et al.’s result and ours are consistent, because both results indicate a rupture plane striking nearly N-S. The differences are probably due to the use of different kinds of data and velocity models in the inversion process.

Events 3, 4, 7, and 8 are earthquakes of lower magnitude (see Table 1); therefore the results of the inversions are less reliable. However, even with the limited quality of the data, the results are consistent with the general pattern outlined by the better-recorded earthquakes.

**Fig 6:**
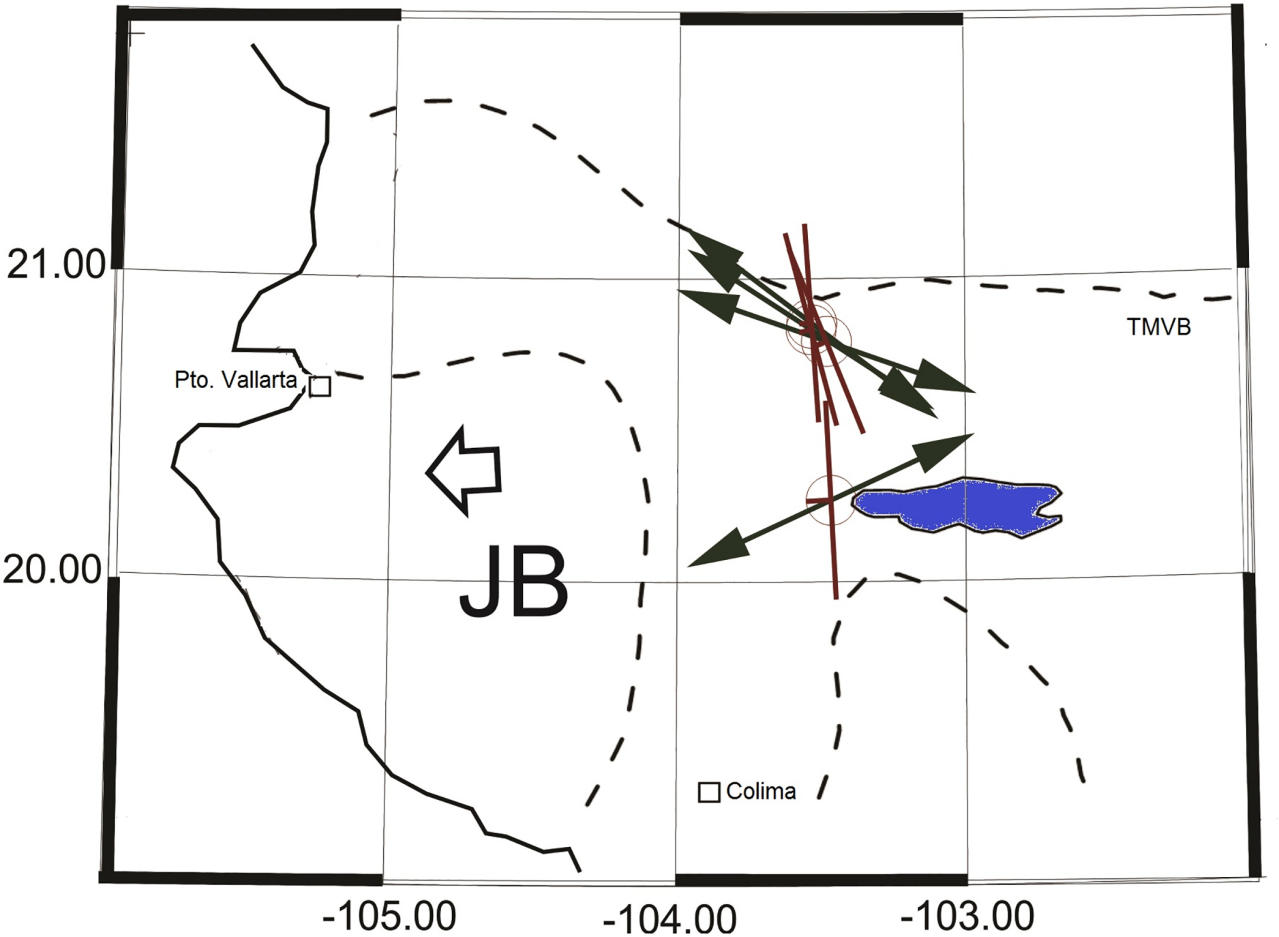
Tension axis map. T-axes of the earthquakes analyzed are shown with black arrows. The preferred nodal planes are shown with red lines. The large arrow marks the approximate direction of the JB motion.

In Fig 6, we have plotted the resulting tension axes and fault planes, assuming that Plane 1 in Table 1 is accepted as rupture plane, for all the earthquakes analyzed. The pattern shows a nearly N-S trending rupture plane. All earthquakes show a normal fault mechanism.

In the same figure, we also plotted the direction of the tension (T-axis) for each earthquake. The earthquakes that cluster northwest of Guadalajara show a consistent direction NW-SE, whereas Event 1 shows a NE-SW direction. The overall data indicate the existence of a band of tension with direction nearly N-S that roughly coincides with the BJ-TMVB contact boundary. The large extent of the affected area of historical earthquakes and the N-S alignment of the ongoing seismicity suggest that the causative mechanism must involve a structure much larger than the mapped local faults.

## Discussion and conclusion

The recent seismic activity in the Guadalajara area initiated with an earthquake M 4.5 on 18 May 2012 (03:07 UT) occurred in the western margin of Lake Chapala. Afterward, the activity migrated 60 km to the northwest of the Guadalajara metropolitan area in 2015. In the group of earthquakes, two, with magnitudes of 4.5 and 4.8 (Events 1 and 6), stand out. These earthquakes, as mentioned before, were widely felt, and minor damage in Guadalajara and other nearby towns was reported.

All the events analyzed show a normal faulting mechanism with a rupture fault plane striking nearly in the direction N-S and mostly dipping about 44°–73° toward the west. The northern cluster of events (Events 2, 5, and 6) have a prominent right-lateral component. Event 1 in the south is a nearly pure normal faulting with a small left lateral component. The distribution of tension axes for the earthquakes shows a preferably NW direction consistent with the proposed motion of the JB toward the west as it rifts from the North America Plate, as derived from other sources of information [24].

The region under study is very complex, and many fault systems have been mapped on geological surveys and satellite image observations in central Mexico [15, 7], a fact that complicates the association of seismicity to a particular fault system. The overall seismicity observation, however, indicates the existence of two seismic regimes in the region. One, larger in extent and probably deeper, with orientation N-S, is probably responsible for the major earthquakes (1568 and 1875) that occurred in the area, which were characterized by a long recurrence time. A second seismic regime that manifests itself through recurrent upper-crust shallow earthquake swarms and sequences, with a smaller ruptures extent and, consequently, minor-magnitude earthquakes, such as the one reported in this article, can be associated to the activation of local fault systems fueled by the interaction of the two main geological structures present in the region: the JB and the TMVB. The results presented are consistent with this conclusion and with the proposed westward motion of the JB as it separates from the North America Plate.

## Supporting information

S1 Fig May 18 2012 earthquake inversion-polarity fault mechanism. (JPG)

S2 Fig May 11 2016 earthquake inversion-polarity fault mechanism. (JPG)
