# Supplementary figures and images for: The origin of the recent (2012–2016) seismic activity in the Guadalajara, Jalisco, Mexico, area: A block boundary interaction?
Source: PLoS One. 2018 Aug 30;13(8):e0200991. doi: 10.1371/journal.pone.0200991 (PMC6116937; doi:10.1371/journal.pone.0200991)

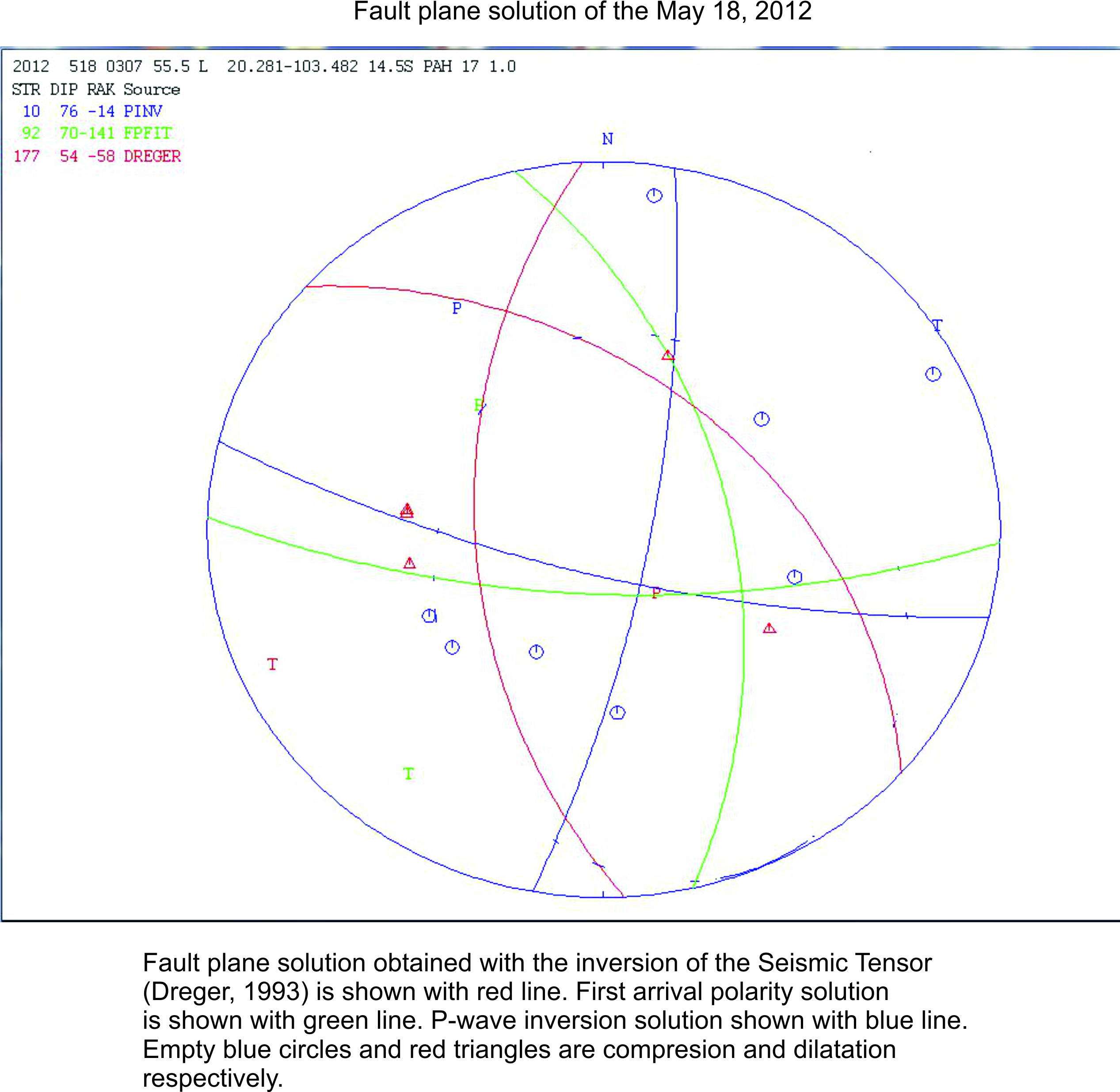

Supplement: S1 Fig — (JPG) [file pone.0200991.s001.jpg]

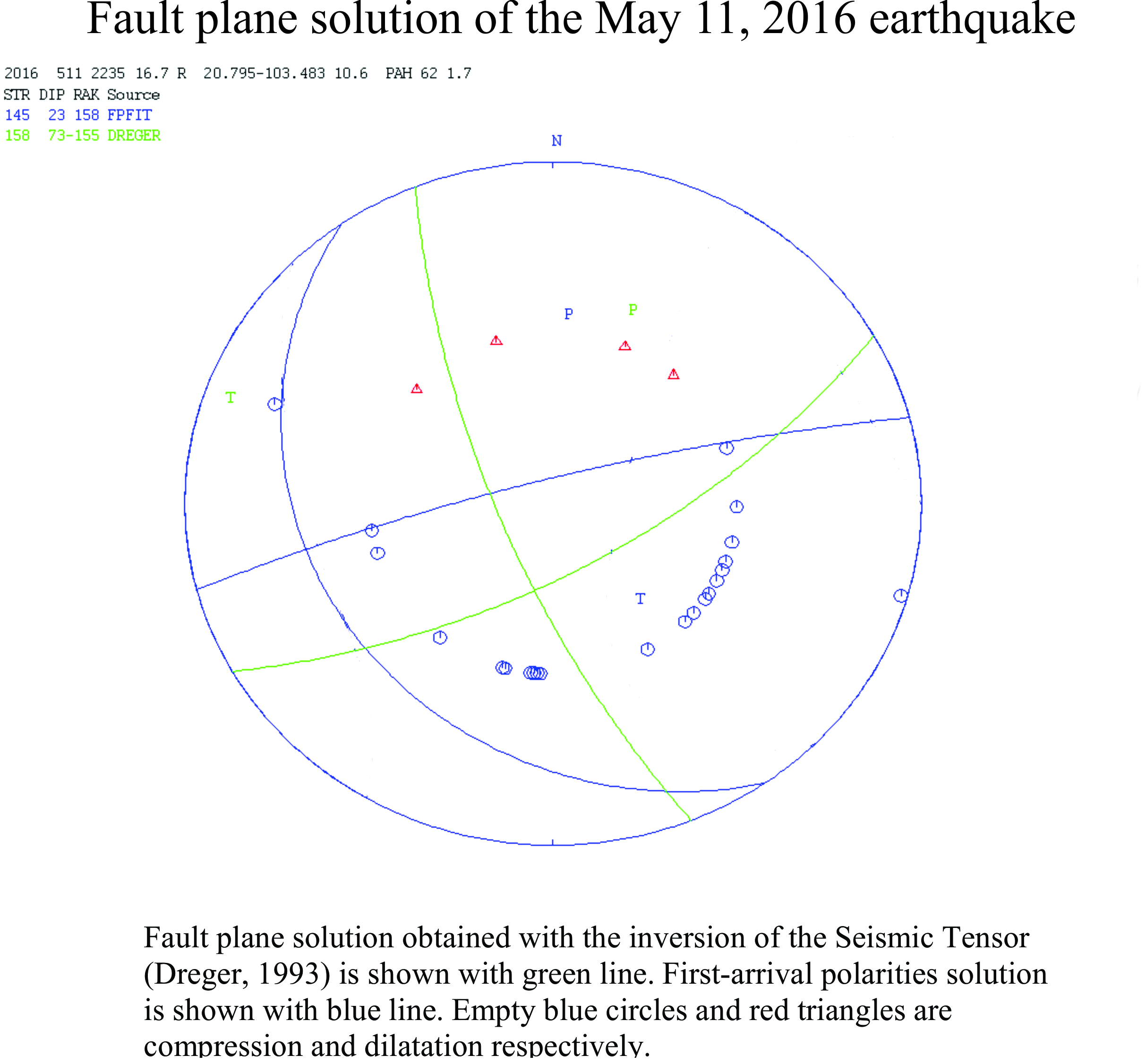

Supplement: S2 Fig — (JPG) [file pone.0200991.s002.jpg]
